# Nanotopographical control of surfaces using chemical vapor deposition processes

**DOI:** 10.3762/bjnano.8.126

**Published:** 2017-06-12

**Authors:** Meike Koenig, Joerg Lahann

**Affiliations:** 1 Karlsruhe Institute of Technology (KIT), Institute of Functional Interfaces (IFG), Hermann-von-Helmholtz-Platz 1, 76344 Eggenstein-Leopoldshafen, Germany; 2 Biointerfaces Institute, University of Michigan (UM), 2800 Plymouth Rd., Ann Arbor, MI 48109, USA

**Keywords:** polymer coatings, polymer structures, structured coatings, vapor deposition polymerization

## Abstract

In recent years much work has been conducted in order to create patterned and structured polymer coatings using vapor deposition techniques – not only via post-deposition treatment, but also directly during the deposition process. Two-dimensional and three-dimensional structures can be achieved via various vapor deposition strategies, for instance, using masks, exploiting surface properties that lead to spatially selective deposition, via the use of additional porogens or by employing oblique angle polymerization deposition. Here, we provide a concise review of these studies.

## Review

### Introduction

Polymer coatings have wide-spread applications, from electronics [1], to sensor systems [2] to biotechnology [3]. The ability to spatially control the surface properties in order to further augment this technological utility has been the subject of intensive research in recent years. In this review, we summarize the work that has been conducted to create patterns and structures using vapor-deposited polymers. Two prominent examples of vapor deposition methods are the thermally activated deposition of poly(*p*-xylylenes) (PPX), as well as plasma-enhanced chemical vapor deposition polymerization, both of which offer many advantages over solution-based deposition methods [4]. Since no solvents are involved, no wetting problems or problems with solvent residues arise, which can potentially interfere with the structuring process. In addition, the process can be applied on thermo- or chemically sensitive substrates and can be used to deposit insoluble polymers. The use of vaporized monomers rather than polymer solutions ensures the conformal coating of the substrate and masks, where required. Many examples exist for the post-deposition structuring of homogeneous coatings, for example, with the use of lithographic techniques [5–7]. Instead, the focus of this review lies on the various methods which can be utilized to form structured coatings during the vapor deposition process.

### Masked deposition

Microstructured masks can be applied to cover parts of the substrate in order to prevent deposition of polymer on these locations [8–9]. Chen and Lahann developed the vapor-assisted micropatterning in replica structures (VAMPIR) method using poly(dimethylsiloxane) (PDMS) masks to pattern reactive PPX derivatives on the surface [10]. Compared to metal masks, the PDMS creates a perfect seal to provide smooth surfaces, avoiding the formation of an air gap between the mask and surface, which results in higher pattern fidelity.

**Figure 1:**
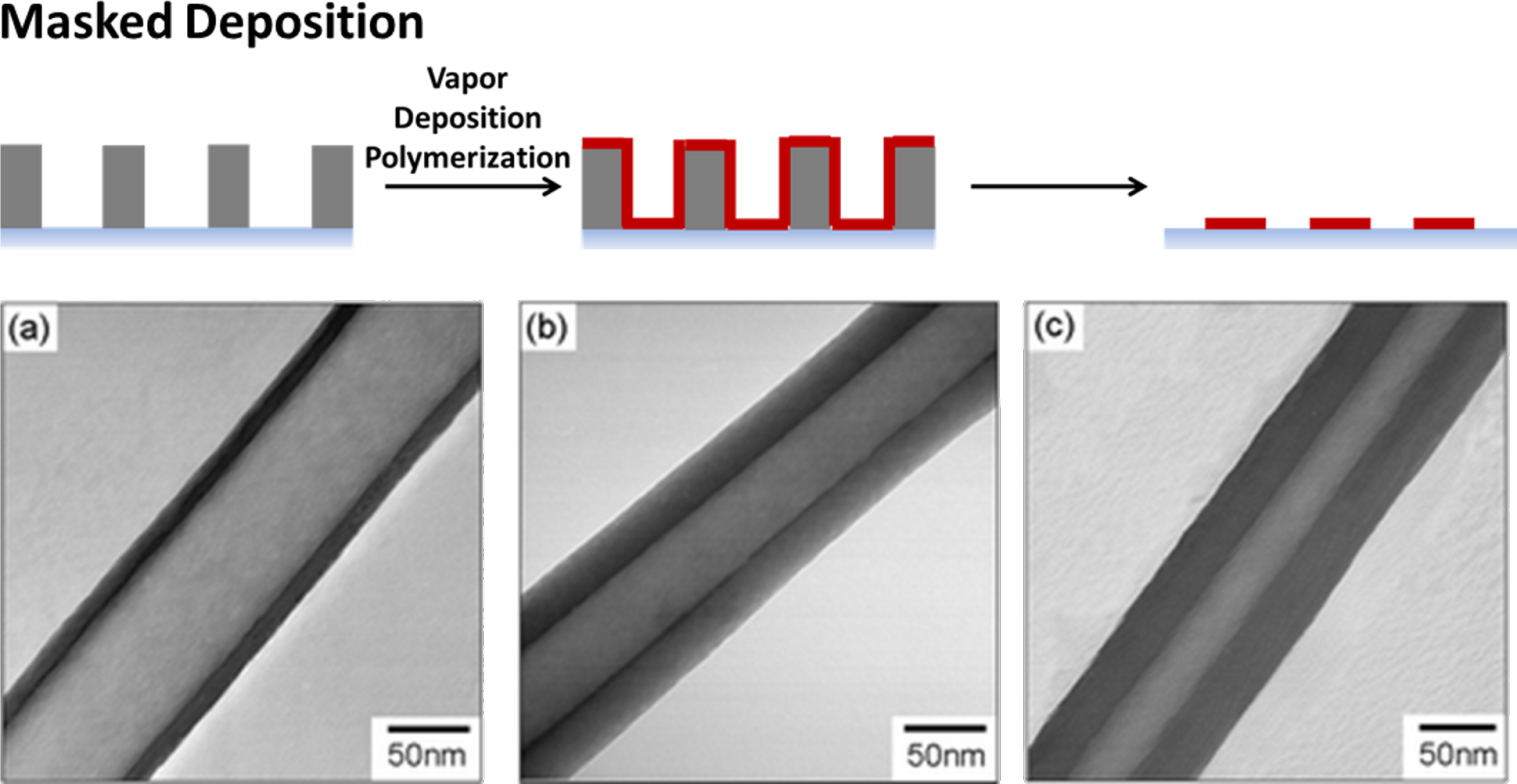
Polymer structures via masked deposition: polypyrrole nanotubes by deposition using aluminum oxide membranes with a pore size of 100 nm as the template. The wall thickness can be varied by variation of the loading amount of monomer (a) 0.07 mL, (b) 0.14 mL, and (c) 0.21 mL. Reproduced with permission from [11], copyright 2004 The Royal Society of Chemistry.

Three-dimensional structures can be formed using masks as well. Jang and Oh used anodic aluminum oxide membranes as a template for the production of nanotubes with a tunable wall thickness (Figure 1) [11]. Via chemical oxidation polymerization of vaporized pyrroles, polypyrrole was deposited on the walls of the membrane pores which had been pretreated with ferric chloride. Membranes with a pore diameter of 20 nm or 100 nm were used. Dissolving the membrane in sodium hydroxide resulted in a solution of nanotubes, which were further carbonized to carbon nanotubes. The wall diameter was controlled to be between 12 to 34 nm by using different amounts of the monomer feed. Trujillo et al. produced polymeric nanostructures using colloidal lithography [12]. In this technique, two-dimensional self-assembled monolayer (SAM) arrays of colloidal nanoparticles serve as lithographic templates for “nanobowl” patterns in an initiated chemical vapor deposition (iCVD) process. The colloidal template was removed by ultrasonication after deposition. Structures derived from a broad range of polymers and across a variety of length scales (down to 25 nm) could be fabricated.

### Selective deposition

A straightforward method to selectively deposit polymers on prepatterned substrates is the structured coating of the substrate by initiator molecules in surface-initiated vapor-deposition polymerization. With this method, the polymer only grows on those locations on the surface equipped with predeposited initiator molecules. The patterning of the initiator can be achieved using photolithography [13], microcontact printing [14] or inkjet printing [15] for instance.

**Figure 2:**
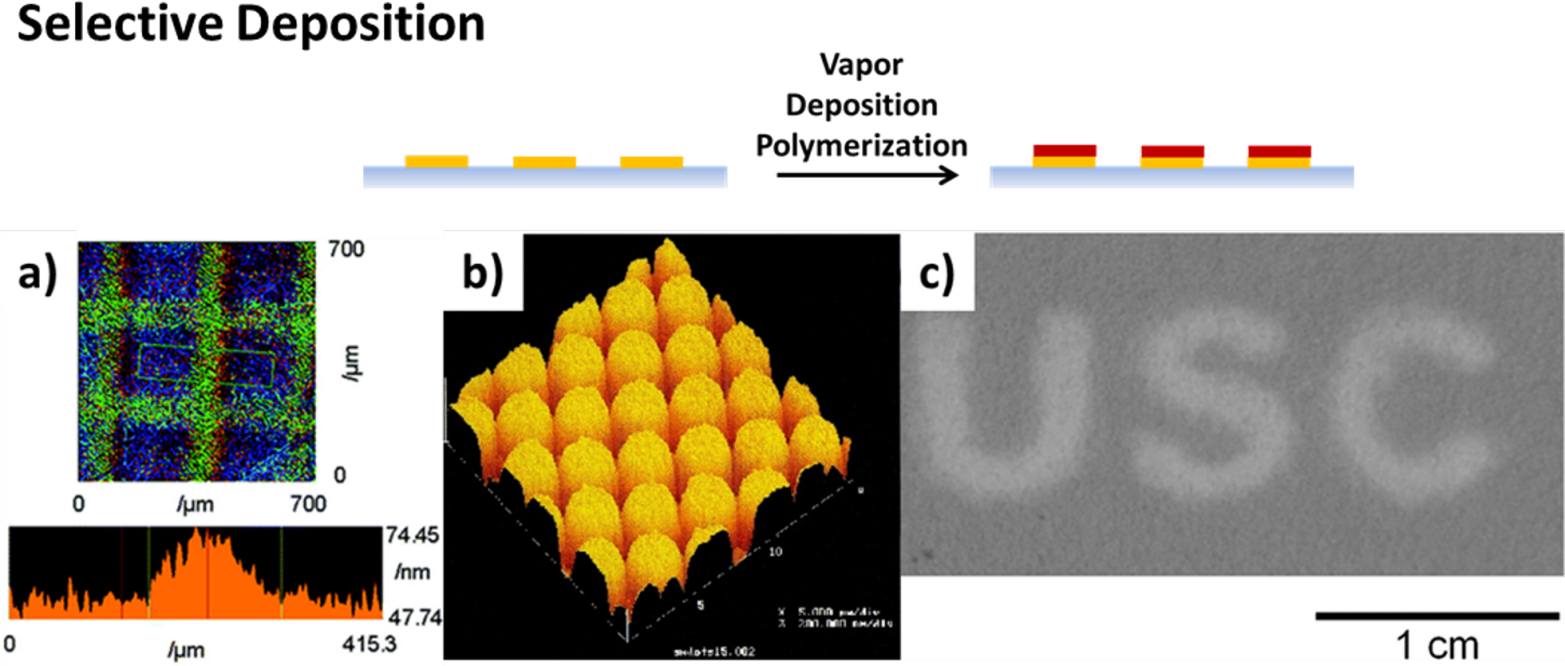
Formation of patterned polymer coatings by selective deposition strategies: a) spatially selective in situ activation of the surface-immobilized initiator in photoinduced vapor-phase-assisted surface polymerization of poly(methyl methacrylate), b) selective deposition of PPX-n on a hexadecanethiol pattern (spot diameter 1.5 µm) on a silver surface, c) selective deposition of poly(4-vinyl pyridine-*co*-divinyl benzene) on copper-free regions (dark area) of chromatography paper. Permissions: (a) was reproduced with permission from [16], copyright 2006 The Royal Society of Chemistry, (b) was reprinted with permission from [18], copyright 2000 American Chemical Society, and (c) was reprinted with permission from [19], copyright 2015, American Vacuum Society.

A second option is the spatially selective in situ activation of the initiator, which has been homogeneously coated on the substrate. Nishida and co-workers created patterns of activated photoinitiator by irradiation of the surface through a mask during the deposition process [16]. In a subsequent report, the use of an auto-drawing system, consisting of an optical fiber irradiation apparatus and a programmed manipulator for the spatially selective activation of the initiator, was demonstrated (Figure 2a) [17].

For PPX, transition metals, as well as their salts or complexes, were found to inhibit the polymer growth on the surface. PPX-n and chlorinated PPX did not grow on metal and metal oxide surfaces. This was likely due to deactivation of the adsorbed bi-radical form, which results in quenching of the chain growth [20]. Due to secondary adsorption on deactivated monomers, the authors found a maximum thickness of selectively grown polymer depending on the metal and the monomer type. Iron was found to be the most efficient inhibitor for the investigated polymers. By patterning iron molecules via photolithography or through a shadow mask, microstructured polymer films were fabricated [21]. Inverted structures were created using microcontact printing of alkanethiols on gold or silver surfaces (Figure 2b). These were found to prevent the quenching effect of the metals, thus promoting polymer growth instead. Using carboxylic acid-terminated alkanethiols, iron salt exposure on the surface could be spatially defined, which again prevented polymer growth [18].

Further investigations with a wider variety of PPX derivatives were conducted by Chen et al. [22]. For PPX derivatives containing oxygen or nitrogen, no inhibition of polymer growth was found on transition metals. For this reason, attractive interactions between the metal and the heteroatoms were suggested. The patterned deposition of a reactive PPX derivative could be realized for PPX–vinyl on titanium, and its reactivity in cross-metathesis reactions was demonstrated.

The inhibition of chain growth by transition metals was also demonstrated for different types of monomers by Kwong et al. [19,23]. Various metals and metal salts were found to inhibit the growth of acrylate-based polymers and poly(4-vinylpyridine) (P4VP). Copper salts such as CuCl_2_ and Cu(NO_3_)_2_ were identified for effective inhibition of all investigated types of polymers. The patterned deposition was demonstrated by screen printing of a solution of the metal salt using a mask. No polymer deposition occurred on locations treated with the metal salt (Figure 2c).

In order to pattern surfaces with PPX derivatives containing nitrogen and oxygen heteroatoms, further work was conducted by Wu et al. [24]. The deposition of various PPX derivatives could be inhibited by electrically charging conducting substrates. Supplying electrical energy to the surface increases the surface energy, which results in the deactivation of the reactive monomer species on the surface. Patterning was realized by placing conducting aluminum metal channels on a nonconductive glass surface [24].

The selective growth of various polymers was also found for surfaces equipped with different functional groups. This results in different adhesion properties of the monomer on the surface. Tsukagoshi et al. used different aminosilanes for locally activating the deposition of polyamide on silicon substrates [25]. On gold surfaces, SAMs of alkanethiolates offer a facile way to supply the surface with various functional groups. Choi et al. directed the growth of poly(isobenzofuran) by patterns of SAMs with different terminal groups [26]. Methyl-terminated SAMs were found to hinder the growth of polymers, while the polymer preferentially grew on carboxylic acid-terminated SAMs. Bally-Le Gall et al. noted selective growth of PPX derivatives with trifluoroacetyl or chlorine functionality on carboxylic acid- or hydroxyl-terminated alkanethiolates as compared to methyl-terminated SAMs [27]. In this way, free-standing nanosheet membranes were fabricated.

### Substrate-induced morphology control

**Figure 3:**
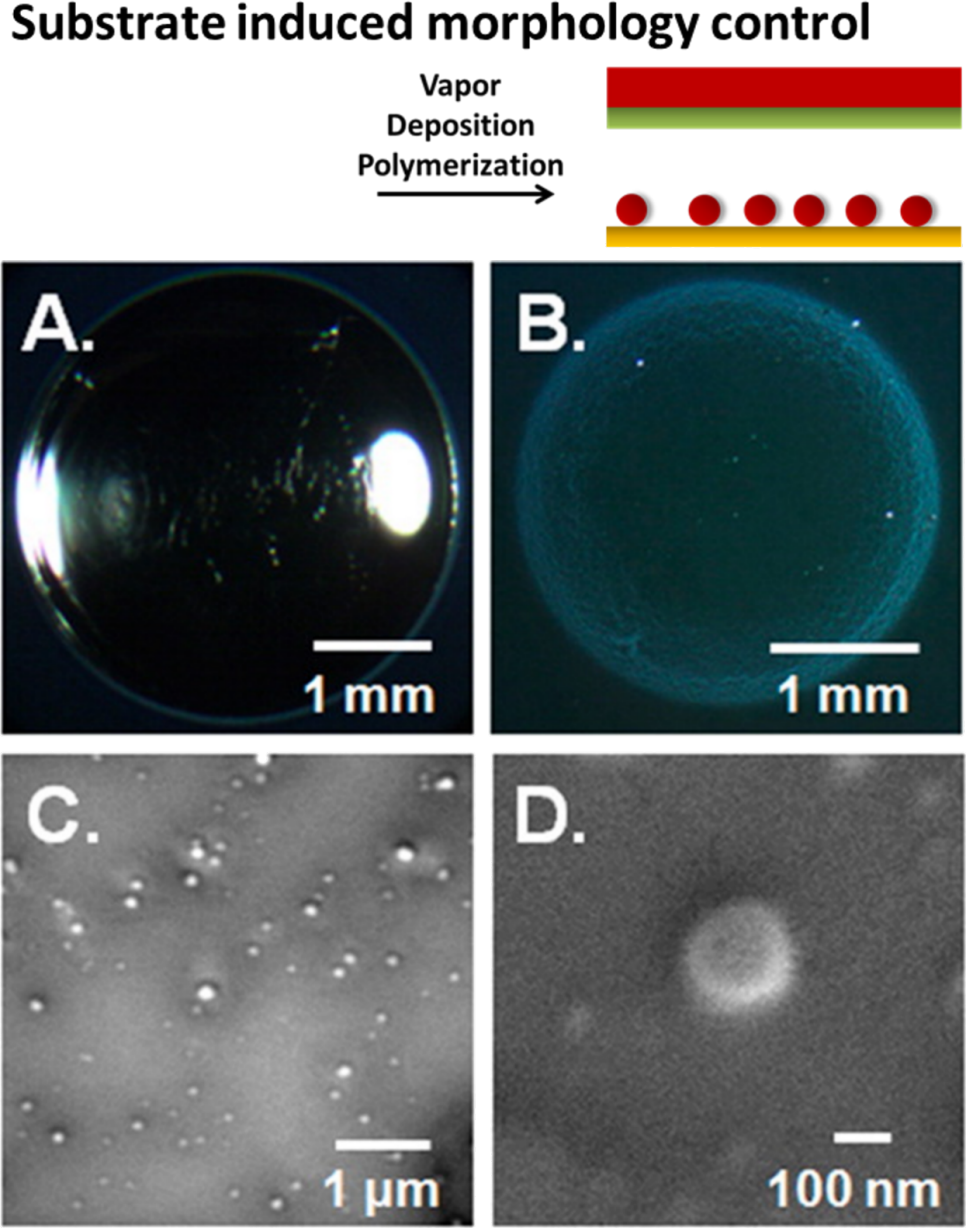
Variation of the polymer structure induced by the substrate: (A) polymer film formed when the spreading of the polymer on the liquid is energetically favorable, poly(1*H*,1*H*,2*H*,2*H*-perfluorodecyl acrylate) on 1-ethyl-3-methylimidazolium tetrafluoroborate, (B) polymer particles formed under energetically unfavorable spreading conditions (poly(4-vinylpyridine) on silicone oil), and (C,D) scanning electron images of poly(4-vinylpyridine) polymer particles. Reprinted with permission from [29], copyright 2013 American Chemical Society.

Demiryürek et al. developed a method to produce periodic wrinkle structures on the surface of polymer films using prestrained substrates [28]. Various polymers were deposited on prestrained PDMS substrates using iCVD. The subsequent release of the strain leads to microstructured wrinkles, where the topography is controlled by tuning the elastic modulus of the polymer coating and the substrate. Haller et al. investigated the morphology of vapor-deposited polymers on liquid substrates (Figure 3) [29]. Depending on surface tension, liquid viscosity, deposition rate and deposition time, either film or particle formation was found. Particles tend to form if the surface tension interaction between the liquid and the polymer is energetically unfavorable, promoting aggregation of the polymer. If the interaction is ambiguous, particle formation is observed at low deposition rates and times and with low liquid viscosity.

### Introduction of porogen during deposition

**Figure 4:**
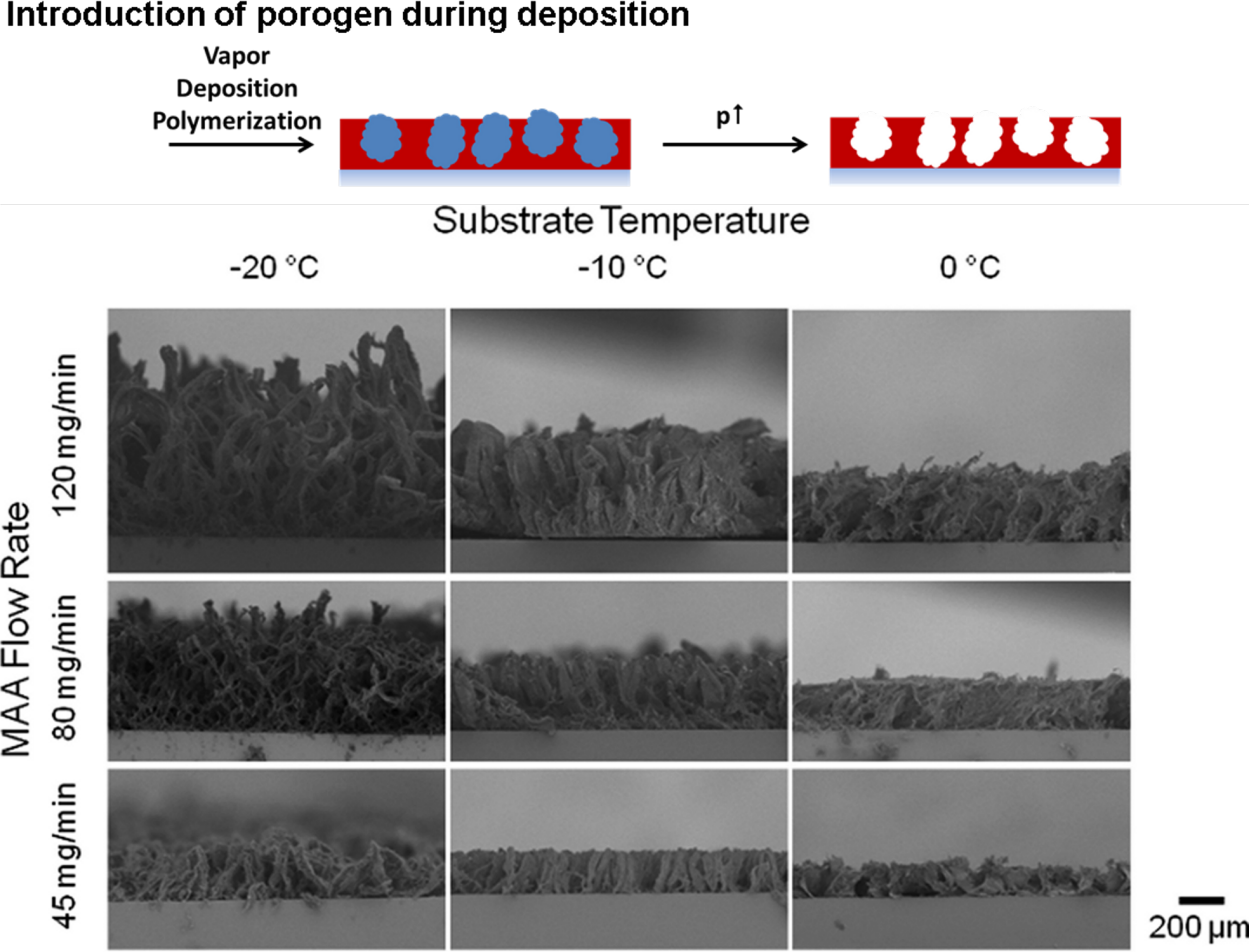
Polymer structures created via introduction of a porogen during the deposition process: the images show the extent of the deposition of solid monomer, which occurs simultaneously with polymerization. Thereby, the resulting morphology can be varied by the monomer flow rates or the substrate temperature. Reprinted with permission from [33], copyright 2014 American Vacuum Society.

Polymer films with porous morphology can be created via the introduction of a porogen into the growth process. Tao and Anthamatten formed open-cell, macroporous poly(glycidyl methacrylate) structures using ethylene glycol as a porogen [30–32]. The inclusion of an inert, condensable species into the gas feed mixture ensures phase separation simultaneously with the polymerization and crosslinking reactions. The porogen is removed in a post-deposition process using vacuum or solvent treatment. Gupta and co-workers demonstrated that in the iCVD process the monomer itself can act as a porogen if unconventional iCVD processing conditions are employed (Figure 4) [33–36]. Increasing the partial pressure of the monomer above its saturation pressure and decreasing the substrate temperature below the freezing point of the monomer results simultaneously in the deposition of solid monomer and polymerization. Following the deposition process, the solid monomer is removed via sublimation, leading to membrane structures with dual-scale porosity. The growth rate and the pore size of the resulting membrane can be controlled by the reactor parameters, such as deposition time, monomer partial pressure and substrate temperature. The three-dimensional growth of pillared microstructures was found at low substrate temperatures, while at increased substrate temperatures, web-like growth occurred. The membrane formation could be spatially controlled by patterning of the surface energy of the underlying substrate using a fluorinated polymer. Minimal nucleation of monomer was found on the fluorinated spots, which led to a dense polymer coating on these sites. These techniques show great promise in the fabrication of membranes [37].

### Oblique angle deposition

**Figure 5:**
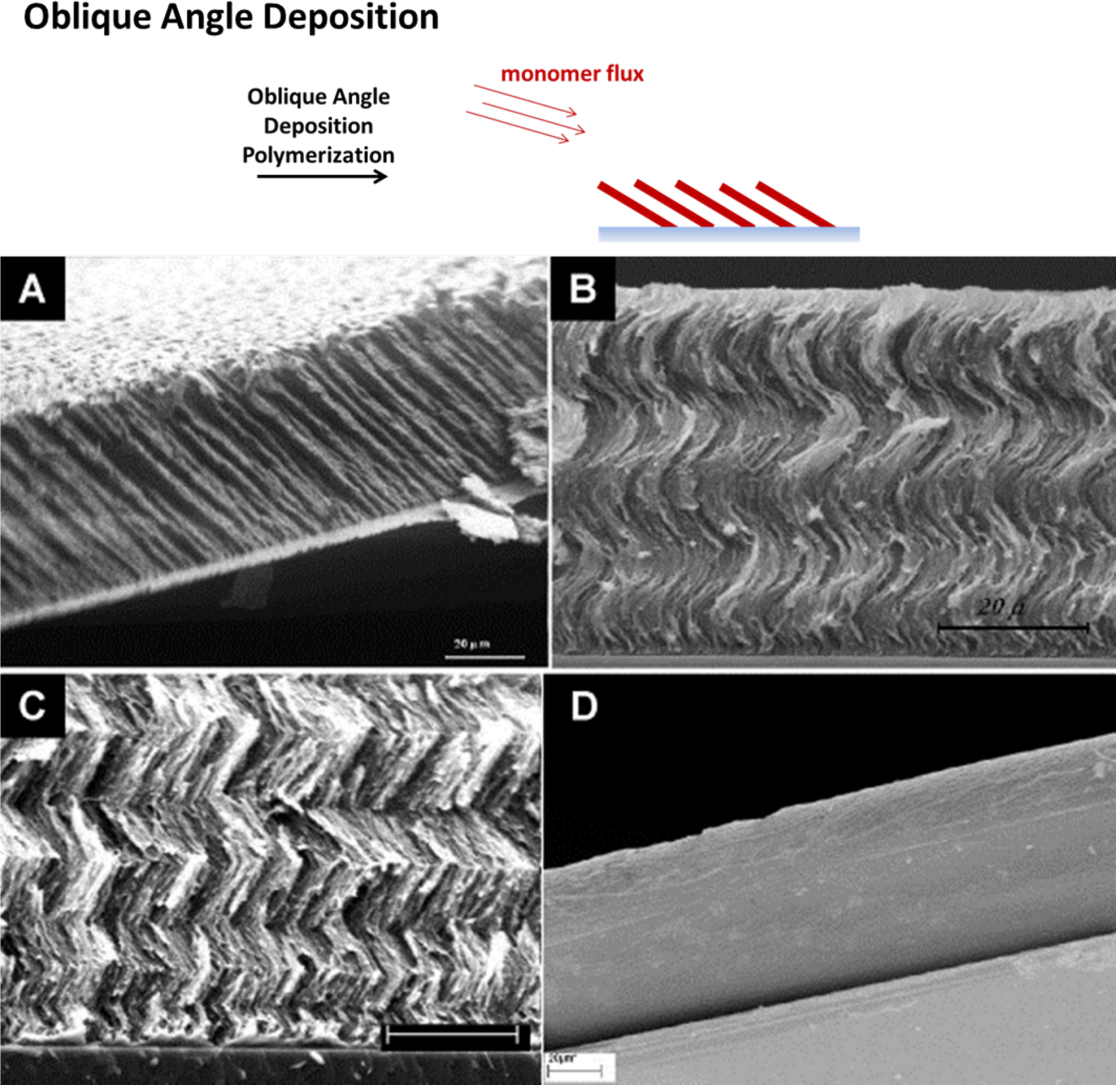
Polymer structures via oblique angle polymerization: by manipulating the substrate rotation during the deposition, complex architectures such as A) columnar, B) helical or C) chevron patterns can be achieved. In contrast to this, D) displays the corresponding cross-sectional scanning electron micrograph of a planar PPX-n film. All scale bars are 20 μm. Reprinted with permission from [40], copyright 2008 Elsevier.

A significant amount of work was conducted by Demirel and co-workers on the formation of 3D polymer structures using oblique angle polymerization deposition, analogous to the method already widely applied for the formation of inorganic structures [38]. The direction of the monomer vapor flux at an oblique angle of around 10° to the substrate plane results in the formation of slanted nanocolumns via a self-shadowing mechanism with a diameter of around 150 nm [39–41]. The slanting angle can be controlled via the deposition angle. Compared to inorganic oblique angle deposition, more complex algorithms have to be applied in order to successfully predict the growth morphology of the polymer structures, taking into account the chain growth kinetics. The morphology can be further varied by manipulating the substrate rotation, resulting in more complex architectures, such as helices or chevron structures (Figure 5). The nanostructured PPX films produced exhibit a high water contact angle and the use in water droplet transport was demonstrated [42–43]. Further potential applications include biomaterial design [44] and catalytic devices [45].

## Conclusion

Vapor deposition polymerization techniques have been successfully applied in order to create patterned and structured polymer coatings. Structuring both in two and three dimensions can be achieved by either using masks or taking advantage of selective deposition properties on prepatterned substrates, exploiting substrate properties such as the surface energy, polarity or interaction with adsorbing monomers. Additionally, the introduction of porogens during the deposition process and the deposition at an oblique angle are methods that have been reported to lead to the formation of three-dimensional polymer structures. With these bottom-up approaches, structured and patterned coatings from a great variety of polymer materials can be created that can be tailored to the respective applications.
